# Neural correlates of affective contributions to lexical decisions in children and adults

**DOI:** 10.1038/s41598-020-80359-1

**Published:** 2021-01-13

**Authors:** Teresa Sylvester, Johanna Liebig, Arthur M. Jacobs

**Affiliations:** 1 Department of Education and Psychology, Experimental and Neurocognitive Psychology, Freie Universität Berlin, Habelschwerdter Allee 45, 14195 Berlin, Germany; 2 Center for Cognitive Neuroscience Berlin, Freie Universität Berlin, 14195 Berlin, Germany

**Keywords:** Cognitive neuroscience, Emotion

## Abstract

The goal of the present study was to investigate whether 6–9-year old children and adults show similar neural responses to affective words. An event-related neuroimaging paradigm was used in which both age cohorts performed the same auditory lexical decision task (LDT). The results show similarities in (auditory) lexico-semantic network activation as well as in areas associated with affective information. In both age cohorts’ activations were stronger for positive than for negative words, thus exhibiting a positivity superiority effect. Children showed less activation in areas associated with affective information in response to all three valence categories than adults. Our results are discussed in the light of computational models of word recognition, and previous findings of affective contributions to LDT in adults.

## Introduction

From a modular perspective of mental functions, language and affect should be processed independently^1^. As a matter of fact, for many decades research on language and emotion progressed basically without much interaction^2^. However, in the last decades the modular perspective was replaced by a distributed interactive one^3–5^, resulting in numerous studies interested in both language and emotion. Nonetheless, it is still an open question how these systems interact, and how linguistic and affective information is connected in performing higher-level cognitive tasks, such as word recognition or lexical decision. While accumulating evidence points to an important role of affective information in word recognition and reading tasks^2,6–12^ performance in the LDT could, in principle, be based on shallow orthographic-phonological processing^13,14^. However, behavioural, brain-electrical (EEG) and neuroimaging (fMRI) evidence clearly indicates that affective semantic information, such as the valence and arousal features of single words, is also used in the LDT^15–18^.

Briesemeister et al.^16^ described an emotion network activated during an LDT involving the anterior cingulate cortex (ACC)^19,20^, posterior cingulate cortex (PCC), insula, hippocampus, amygdala and orbitofrontal cortex (OFC)^21^ indicating an important contribution of affect in lexical decisions. Furthermore, Ponz et al.^11^ and Ziegler et al.^12^ found anterior insula activation in response to disgusting words, whereas Lindquist et al.^22^ examined different valence approaches and found anterior insula as a general hub for valence processing. Kuhlmann et al.^23^ presented neuroimaging evidence for left inferior frontal gyrus (lIFG) involvement during valence decisions proposing that on top of many other functions^24–28^ it is also an integrative hub for affective semantic information.

All above-mentioned results are based on data from adults and much less is known about affective semantic processing in children. In particular, it is still unknown whether children also rely on affective semantic information to make lexical decisions. Usually studies investigating lexical processing in children only use behavioural data and ignore affective contributions^29,30^ (except^31,32^). Weiss-Croft and Baldeweg^33^ summarised fourteen neuroimaging studies that focused either on phonological or semantic decisions in 7-year old children up to young adults. Similar to the behavioural studies, all these studies did not specifically examine the neural correlates of emotional aspects of language but rather focused on cortical structures while ignoring potential subcortical contributions. A common finding was an increased activation in the left ventral language network with age, i.e. in left superior and middle temporal gyrus. Weiss-Croft and Baldeweg^33^ related this age effect to an ongoing growth of the mental lexicon with exposure to language and thus, richer semantic representations with increasing competition between them. They also identified increasing activity in IFG and supramarginal gyrus, associated with semantic and phonological decision making. There is an ongoing discussion whether or how the language system in the brain changes due to cumulative exposure to language^33^ or whether it is already relatively stable after the age of six^34^. Despite functional fine-tuning during development, children’s language system shows remarkable similarities to the adult system already at the age of six^34^. To answer the question whether this is also true for affective contributions to word recognition is the goal of the present study.

### Present study

We investigated affective semantic contributions to word recognition in 6–9-year old children and 19–30 year old adults. To avoid potential confounding effects of reading effort (especially in children), stimuli were presented auditorily. Sylvester et al.^32^ had already shown that visual and auditory word presentation led to equivalent behavioural results in a valence rating task (see also^35^). Also, on the neural level, Price^28^ described only small differences in lexico-semantic processing in the visual and auditory modality. In the present study, all participants performed the same auditory LDT in the fMRI scanner using positive, negative and neutral words as well as pseudowords. We expected similarities of lexical processing in children and adults based on former findings e.g. in left middle frontal regarding semantic retrieval and superior temporal due to auditory processing^28,33^. We also expected affective contribution facilitating the lexical decisions as found for adults^15,16,18^. For children, however, affective-semantic contributions to lexical decisions are less well studied and thus, predictions are not as clear. If activations are similar to adults, they will encompass lIFG, OFC, ACC, PCC, hippocampus and amygdala^15,16,18^.

## Results

### Behavioural results

On the behavioural level, reaction times were analysed by calculating the mean times between stimulus presentation and button press by one factorial ANOVA and pairwise t-tests between valence categories. Only trials with correct responses entered analysis. Adults showed clear reaction time effects of word valence *(F(3, 1794)* = *43.41, r*^*2*^ = *0.06)*, with the shortest reaction times for positive words *(M* = *424 ms, SD* = *19 ms)* followed by negative words *(M* = *466 ms, SD* = *20 ms)* and neutral words *(M* = *472 ms, SD* = *21 ms)*. Reaction time differences between positive and neutral words were statistically significant (*t* = *− 1.68, p* < *0.05*). The longest reaction times were observed for pseudowords *(M* = *636 ms, SD* = *11 ms)* which were significantly longer than for all three valence categories (pseudowords—positive words: *t* = *9.37, p* < *0.0001*; pseudowords—negative words: *t* = *7.52, p* < *0.0001*; pseudowords—neutral words: *t* = *6.89, p* < *0.0001*). Almost the same reaction time pattern was observed for children *(F(3,996)* = *15.04, r*^*2*^ = *0.04)*. Here, the three valence categories showed no significant differences but still reaction times for positive words were shortest *(M* = *693 ms, SD* = *29 ms)* followed by neutral words *(M* = *698 ms, SD* = *29 ms)* and negative words *(M* = *747 ms, SD* = *30 ms)*. All three valence categories had significantly shorter reaction times than pseudowords *(M* = *873 ms, SD* = *17 ms;* pseudowords—positive words: *t* = *5.32, p* < *0.0001;* pseudowords—negative words: *t* = *3.61, p* < *0.0001;* pseudowords—neutral words: *t* = *5.12, p* < *0.0001)*.

### MRI results

For comparison with previous studies using auditory LDT, we first computed the contrast word > pseudowords. We then looked directly at the valence categories activations during the auditory LDT.

### Words (positive, negative, neutral) > pseudowords

**Figure 1:**
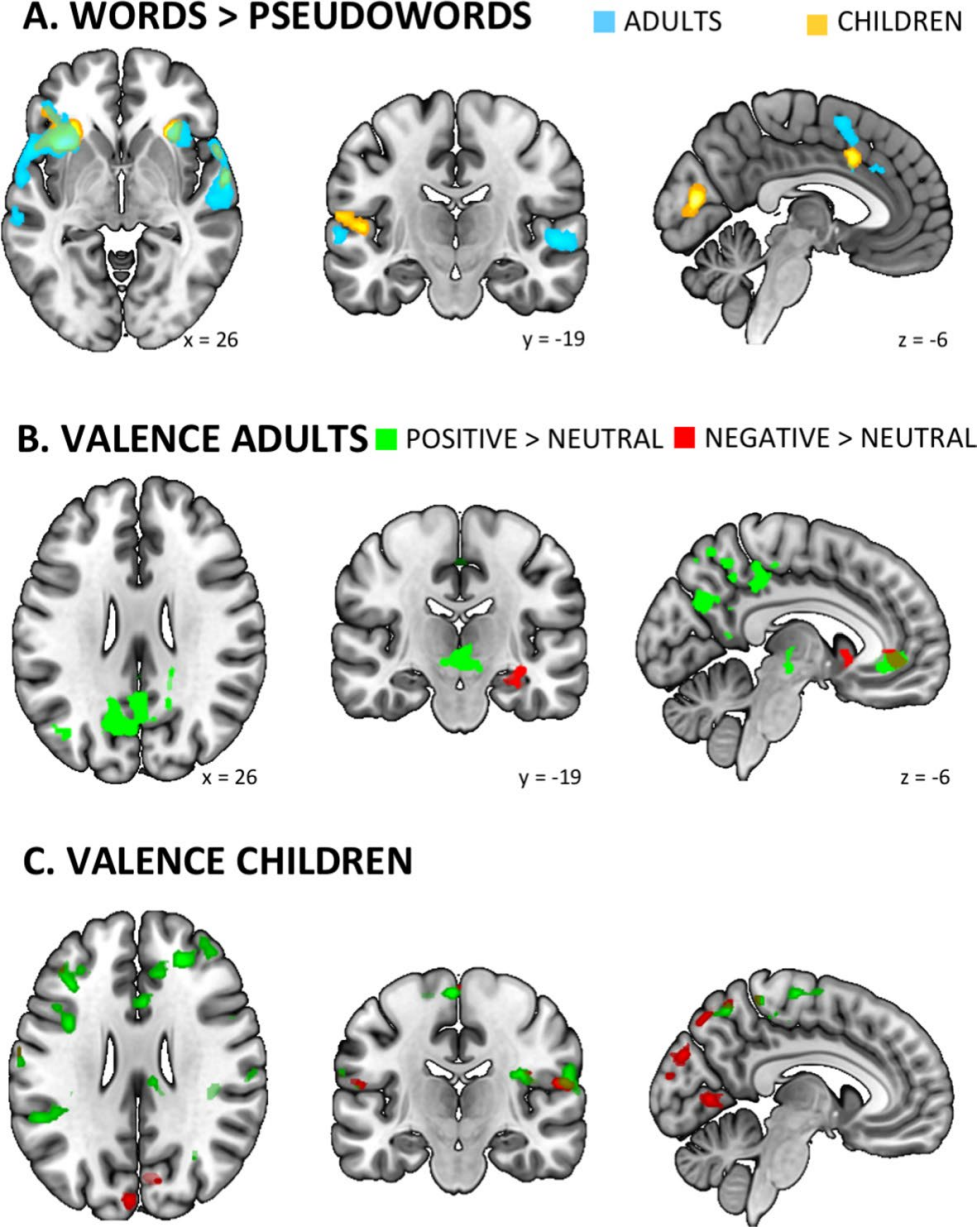
(**A**) Blood oxygen level dependent (BOLD) response showing greater activation for words compared to pseudowords in adults (blue) and children (yellow) and their overlap (green). (**B**) BOLD response in adults for the contrast positive compared to neutral words in green and for the contrast negative compared to neutral words in red. (**C**) BOLD response in children for the contrast positive words compared to neutral words in green and for the contrast negative compared to neutral words in red.

Adults showed increased left hemispheric middle frontal activation including superior frontal and supplementary motor area (SMA) activation including right dorsal cingulate in response to words compared to pseudowords. Increased right hemispheric activation was observed in superior temporal including temporal pole and additionally the bilateral anterior insula including lIFG reached significance (see Table 1, Fig. 1A in blue).

**Table 1:** Adults’ activation for the contrast words (positive, negative, neutral) compared to pseudowords (FWE corrected, p < 0.05, cluster level).

**Anatomical location**	**MNI**			**Size**	**Peak**
	**x**	**y**	**z**	**k**	**T**
**Frontal**					
L Middle frontal	− 30	52	16	209	5.22
* L Superior frontal*	− 26	56	8		4.37
**Subcortical structures**					
L Insula anterior	− 38	16	− 2	1369	6.12
* L IFG*	− 46	8	12		5.24
R Insula anterior	34	26	− 4	222	4.58
**Temporal**					
R Superior temporal	64	− 8	0	679	7.13
* R Superior temporal pole*	56	16	− 8		6.17
**Supplementary motor area**					
L SMA	2	12	48	470	5.29
* R Dorsal cingulate*	4	12	38		4.84

*x, y, z* peak coordinates according to MNI stereotactic space, *k* cluster size in voxels, *T*-values for peaks, *R * right, *L* left, *IFG* inferior frontal gyrus, *SMA* Supplementary motor area.

Children showed significantly higher left hemispheric activation in middle frontal areas including lIFG, dorsal cingulate including right SMA and dorsal cingulate, as well as planum temporale including superior temporal. Right hemispheric activation in superior temporal areas including superior temporal pole was significantly increased as well as bilateral activation in the anterior insula including right hemispheric medial OFC and calcarine (see Table 2, Fig. 1A in yellow).

**Table 2:** Activation in children for the contrast words (positive, negative, neutral) compared to pseudowords (FWE corrected, p < 0.05, cluster level).

**Anatomical location**	**MNI**			**Size**	**Peak**
	**x**	**y**	**z**	**k**	**T**
**Frontal**					
L Middle frontal	− 28	54	16	130	4.56
* L IFG*	− 32	38	12		3.55
**Subcortical structures**					
L Insula anterior	− 34	18	− 2	776	5.99
R Insula anterior	30	26	0	216	5.47
* R Orbitofrontal*	0	12	− 12		3.78
L Dorsal cingulate	0	12	40	234	5.27
* R SMA*	6	22	36		4.33
* R Dorsal cingulate*	10	8	36		3.74
**Temporal**					
L Planum temporale	− 62	− 28	10	632	4.42
* L Superior temporal*	− 66	− 34	14		4.37
R Superior temporal	62	0	− 6	134	4.95
* R Superior temporal pole*	60	10	− 6		4.44
**Occipital**					
L Calcarine	− 8	− 82	10	586	5.8
* R Calcarine*	10	− 78	10		5.57

*x, y, z* peak coordinates according to MNI stereotactic space, *k* cluster size in voxels, *T*-values for peaks, *R * right, *L* left, *IFG* inferior frontal gyrus, *SMA* supplementary motor area.

At first glance results of adults and children seem very similar. Thus, a conjunction analysis was performed showing that adults and children shared activation for the contrast words (positive, negative, neutral) > pseudowords in left anterior insula (*k* = *440, T* = *5.99*, [− 34 18 − 2]) including lIFG (*T* = *3.76*, [− 42 36 − 4]).

### Valence-specific effects in adults

For the contrast positive > neutral words, adults showed activations in the left hemisphere in precentral gyrus including PCC, ACC including right superior frontal, angular gyrus including middle occipital gyrus and precuneus. Additionally, the left ventral diencephalon (vDC) including right vDC reached significance.

For the contrast negative > neutral words increased activations were found in left medial OFC including caudate and putamen, left hippocampus including fusiform and in right ACC including superior frontal (see both contrasts in Table 3, Fig. 1B).

**Table 3:** Adults’ activation for the contrasts positive words compared to neutral words and negative words compared to neutral words (FWE corrected, p < 0.05, cluster level).

**Anatomical location**	**MNI**			**Size**	**Peak**
	**x**	**y**	**z**	**k**	**T**
**Positive > neutral words**					
**Frontal**					
L Precentral	0	− 26	48	1036	6.23
* L PCC*	− 4	− 36	42		6.03
**Subcortical structures**					
L ACC	− 6	44	− 4	512	5.81
* R Superior frontal*	8	44	− 4		5.29
L vDC	− 4	− 20	− 8	163	5.57
* R vDC*	10	− 22	− 8		4.85
**Parietal**					
L Angular	− 46	− 68	20	151	4.94
* L Middle occipital*	− 38	− 80	28		3.69
L Precuneus	− 14	− 68	28	689	6.67
**Negative > neutral words**					
**Frontal**					
L Medial OFC	− 20	34	− 10	247	5.97
* L Caudate*	− 10	14	− 4		4.81
* L Putamen*	− 20	20	− 8		4.76
**Subcortical structures**					
L Hippocampus	− 34	− 18	− 10	221	5.74
* L Fusiform*	− 42	− 16	− 20		4.86
R ACC	6	44	− 4	854	6.89
* R Superior frontal*	20	50	2		6.73

*x, y, z* peak coordinates according to MNI stereotactic space, *k* cluster size in voxels, *T*- values for peaks, *R * right, *L* left, *PCC* posterior cingulate cortex, *ACC* anterior cingulate cortex, *vDC* ventral diencephalon, *OFC* orbitofrontal cortex.

The conjunction analysis for both, positive > neutral and negative > neutral words showed activations in left ACC *(k* = *278, T* = *5.36, [*− *6 44 *− *4])* including right superior *(T* = *5.29, [8 44 *− *4])* and medial *(T* = *4.15, [2 48 *− 10*])* frontal gyrus.

The direct comparison of both valence categories was significant only in the differential contrast positive > negative in left angular gyrus *(k* = *303, T* = *4.28, [*− *32 *− *72 44])* including superior parietal gyrus *(T* = *3.89, [*− *26 *− *60 46])*.

### Valence-specific effects in children

For the positive > neutral words contrast activations were found in bilateral middle frontal gyrus including right ACC and bilateral IFG and right anterior insula. In the right hemisphere, activation of planum temporale and lingual gyrus including left precuneus reached significance. In the parietal lobe, neural activity was higher for positive than neutral words in the bilateral superior parietal including left angular gyrus as well as right supramarginal and postcentral gyrus including left precentral gyrus.

For negative > neutral words activations were found in the left hemisphere, specifically in the middle frontal and middle temporal areas including inferior temporal and middle occipital areas. Further activations were found bilaterally, i.e. in superior parietal gyrus (including left precuneus), supramarginal, postcentral, lingual and cuneus (see both contrasts in Table 4, Fig. 1C).

**Table 4:** Children’ activation for the contrasts positive words compared to neutral words and negative words compared to neutral words (FWE corrected, p < 0.05, cluster level).

**Anatomical location**	**MNI**			**Size**	**Peak**
	**x**	**y**	**z**	**k**	**T**
**Positive > neutral words**					
**Frontal**					
L Middle frontal	− 38	36	20	468	7.21
L IFG	− 40	2	22	224	4.84
R IFG	40	14	18	702	7.18
* R Insula anterior*	34	8	14		5.91
R Middle frontal	24	40	28	473	5.78
* R ACC*	12	36	26		4.78
**Temporal**					
R Planum temporale	62	− 20	18	288	5.15
**Parietal**					
L Superior parietal	− 24	− 72	54	4086	7.95
* L Angular*	− 44	− 64	16		7.25
R Superior parietal	26	− 48	46	1958	7.31
R Postcentral	6	− 40	66	808	9.63
* L Precentral*	− 4	− 22	66		6.25
R Supramarginal	42	− 30	32	536	7.28
**Occipital**					
R Lingual	8	− 60	2	291	5.63
* L Precuneus*	0	− 64	12		3.71
**Negative > neutral words**					
**Frontal**					
L Middle frontal	− 46	50	6	360	5.49
**Temporal**					
L Middle temporal	− 50	− 62	8	794	6.1
*L Inferior temporal*	− 46	− 48	− 20		5.87
* L Middle occipital*	− 54	− 70	10		5.86
**Parietal**					
L Superior parietal	− 12	− 74	56	527	6.05
* L Precuneus*	0	− 58	56		5.73
L Supramarginal	− 58	− 34	44	723	7.38
R Supramarginal	56	− 36	48	582	6.21
* R Superior parietal*	46	− 46	58		5.5
R Postcentral	6	− 40	66	234	9.01
* R Precentral*	2	− 26	70		4.22
*L Postcentral*	− 8	− 44	64		3.72
**Occipital**					
L Lingual	− 2	− 74	6	586	7.73
* R Lingual*	8	− 62	0		6.06
R Cuneus	6	− 78	30	410	5.65
* L Cuneus*	− 4	− 88	30		5.56

*x, y, z* peak coordinates according to MNI stereotactic space, *k* cluster size in voxels, *T*-values for peaks, *R * right, *L* left, *ACC* anterior cingulate cortex.

The conjunction analysis for positive > neutral and negative > neutral words showed activations in bilateral superior parietal gyrus (left: k = 309, T = 5.94, [− 24 − 72 54], including precuneus, T = 5.46, [0 − 56 54], right: k = 275, T = 5.45, [46 − 46 58]), left hemispheric supramarginal (k = 477, T = 6.81, [− 56 − 34 44]) and middle temporal (k = 437, T = 5.59, [− 48 − 64 10]) and right hemispheric postcentral (k = 164, T = 9.01, [65], including precentral, T = 4.22, [2 − 26 70]) and lingual (k = 193, T = 5.63, [8 − 60 2]) extending into calcarine (T = 3.64, [8 − 60 12]). In both differential contrasts, positive compared to negative and vice versa, the direct comparison of both valence categories led to no significant differences.

### Conjunction analyses of valence-specific effects in children and adults

The conjunction analysis of children and adults regarding positive compared to neutral words showed similar activations in left precuneus (k = 150, t = 5.63, [0 − 56 50]) for both cohorts. The conjunction analysis for negative > neutral words showed no significant shared activations. Further activations regarding all three valence categories (conjunction positive, negative and neutral words) are documented per age cohort and within an age conjunction analysis in Supplementary (A–C).

## Discussion

The present study investigated the question whether children similarly to adults coactivate regions associated with affective semantic processing while performing a standard auditory LDT without any explicit focus on emotion-relevant processing^16,18^. To answer this question, children (6–9-year old) and adults performed the LDT in the scanner with the words’ valence being systematically manipulated. On the behavioural level, our results revealed similar reaction time patterns for children and adults. However, unlike the adults’ data, reaction time differences between positive and neutral words were not significant for children. Both age cohorts showed the shortest reaction times for words and longest for pseudowords. Our behavioural findings mirror previous results indicating a processing advantage for words compared to pseudowords (see lexicality effect^36^) and, within words, a processing advantage for positive words > neutral words as also typically found in adults^37,38^. Thus, we replicated a common finding from visual LDT^39^ in the auditory modality. On the neural level, both age cohorts showed similarities in activation while processing words > pseudowords, especially regarding affective semantic integration processes in anterior insula and lIFG. Concerning valence specific effects, activation in areas associated with affective information was found in both age cohorts. We interpret these findings as further evidence for implicit affective contributions to lexical decisions.

Both age cohorts (see Table 1 for adults and Table 2 for children) showed similar activations contrasting (positive, negative, and neutral words) > pseudowords as already reported in previous studies including the left middle frontal and bilateral superior temporal gyrus as well as SMA. These regions were previously reported in various auditory LDT studies (for adults^40^; for children^33,41^) and are associated with word retrieval^28,42^ and lexico-semantic processing^43^. However, the conjunction analysis of both age cohorts regarding this contrast showed activation in lIFG and insula. Usually lIFG activation is associated with integrative processes of syntactic, semantic and affective information^23,24,26^ while anterior insula is associated with processing of affective information^11,12,44,45^. Both findings are in line with previous studies in adults^15,16^ showing affective semantic contributions to lexical decisions. With the results of the present study these findings are for the first time extended to a cohort of young children.

However, children showed additional activation in bilateral calcarine, dorsal cingulate and superior temporal gyrus. Bilateral calcarine activation is usually associated with auditory semantic tasks^46^ and bilateral superior temporal including left planum temporale activation is associated with early auditory analysis and prelexical processing^28^. Kanske and Kotz^47^ reported dorsal cingulate activation in association with an (emotional) conflict task. Due to enhanced activation in areas associated with prelexical and auditory (semantic) processing and dorsal cingulate activation in children, one can tentatively assume that these mirror children’s effort in performing the LDT.

In general, the investigation of the neural encoding of the lexical status of auditorily presented words indicates that children and adults recruit similar resources. To test this assumption, a conjunction analysis was performed (words children > pseudowords children and words adults > pseudowords adults). The results showed significant activation in left insula extending into the lIFG. We interpret this finding as shared valence processing^11,12,22,44,45^ and similar affective semantic integration processes^23^ irrespective of age or developmental stage.

When looking at valence specific effects (positive > neutral and negative > neutral words), adults (Table 3) showed a more distributed activation pattern for the contrast positive > neutral than for the contrast negative > neutral words. This represents further support for the computational model of word recognition by Hofmann and Jacobs^3^ which assumes more distributed semantic networks for positive than for negative words due to higher cohesion (i.e., more semantic associates^3^). Interestingly, for the contrast positive > neutral words adults (Table 3) and children (Table 4) showed activations usually associated with different stages of phonological processing rather than neural regions classically reported for affective encoding. These include left hemispheric angular gyrus, precentral gyrus and precuneus. Binder et al.^48^ reported angular gyrus activation during a passive listening task, left precentral involvement in phonological decisions and precuneus activation as part of the widely distributed semantic network, involved in encoding of the meaning of speech^28^.

Compared to the results for adults, which showed different response patterns for the contrasts positive > neutral and negative > neutral, children showed more similarities (Table 4). Their neural correlates of affective semantic processing encompass superior parietal, supramarginal gyrus, precuneus and postcentral. Previously, these regions have mainly been associated with semantic and phonological processing^28,34,42^ and, as a common finding, in the auditory LDT^24,38,40^. Interestingly, within the contrast negative > neutral words, most activations were mapped bilaterally. We interpret this as an indicator of task difficulty^49^.

With respect to our initial question about the involvement of affective semantic information during lexical decisions, we indeed found neural responses in several regions usually associated with affective processing as hypothesised. In adults, this holds for the contrast positive > neutral words, i.e. activations in left ACC and PCC. For the contrast negative > neutral words, affective contributions might be reflected in neural activity in left medial OFC and hippocampus as well as right ACC. Since both valence categories elicited activation in left ACC, our findings can be interpreted as further evidence for an implicit affective network including ACC, PCC, OFC, medial temporal gyrus, hippocampus and amygdala, as described by Briesemeister et al.^15,16^. According to these authors, amygdala activation might primarily be associated with arousal. Since we controlled arousal across all three valence categories this could explain why we did not find any amygdala activation.

In children, neural activity was found bilaterally in inferior frontal for positive > neutral words and in middle frontal for negative > neutral words. Similarly to adults, children recruited regions like lIFG including insula and ACC associated with affective information—at least for positive words. On the behavioural level, Kazanas and Altarriba^50^ as well as Yin,Yuan and Zhang^51^ reported a processing advantage of positive stimuli in different modalities and settings in adults, termed the positivity superiority effect^37^. Our behavioural data support this effect, i.e. faster reaction times for positive words in both adults and children, although the difference did not reach significance in the latter.

For the children’s contrast negative > neutral words, no neural region associated with affective information processing reached significance, although it was shown for words > pseudowords. Thus, we analysed similarities in activation of the three valence categories (positive, negative, neutral) via a conjunction analysis (see Supplementary, Table C and D for detailed results). Results showed the contribution of areas associated with affective information processing for all three valence categories in both children and adults, as well as shared regions for both age cohorts (Supplementary, Table E). Thus, processing of neutral words led to activation in areas associated with affective information as well. To examine these differences, further differential contrasts in the opposite direction, namely neutral > positive and neutral > negative, were calculated separately for children and adults. Both contrasts in both age cohorts did not show significant differences. In the context of recent behavioural and computational findings^52^ neutral words also possess some amount of associative affective information. Neutral words are often less arousing than valenced words^59^. In the present study arousal was controlled over all three valence categories, which might have led to an oversampling of ambiguously valenced words with comparatively high arousal values.

In summary, we replicated previous findings in adults and children performing an (auditory) LDT and found the first evidence we are aware of that neural resources associated with affective processing contribute to lexical decisions in children. Compared to the children's data, adults’ data shows more pronounced effects which might be due to their broader, better developed mental lexicon. Consequently, adults can rely on a sophisticated affective semantic network, which facilitates word recognition^3^.

As a tentative explanation of the observed positivity superiority effect^32,37^, we would like to propose a developmental affective semantic hypothesis, according to which the effect reflects an earlier automatic access to positive affective information. Computational modelling of elementary affective decisions^53^ suggests that words’ associations with the positive emotion ‘joy’ play a much stronger role in such decisions than e.g. associations with the negative emotion ‘disgust’. Thus, the present neural response patterns indicate affective contributions to lexical decisions prominent for both valence categories in adults but only for positive words in children. This pattern might reflect the development of the positivity superiority effect. Positive affective information may facilitate faster automatization (integration) processes, which are milestones in the language development of children^33^. However, since this is the first neuroimaging study investigating affective contributions to word recognition in children in a small sample with relatively few stimuli in a single language (German), the results need to be interpreted cautiously and replications on lager samples and extended stimulus materials (e.g., English words with a frequency manipulation as done in adults^54^) should follow.

In the present study, we examined affective semantic processing in young children. Children not only rely on lexical information to successfully solve an auditory LDT but also recruit affective regions when processing positive words, similarly to adults. In sum, we show first evidence for a co-development of affect and language as reflected by the positivity superiority effect.

## Material and methods

### Ethics

The Ethics Committee of the German Association for Psychology (DGPs) and the ethics committee of the Freie Universität of Berlin approved experimental procedure and was in accordance with the principles expressed in the declaration of Helsinki. Informed written consent was obtained from all participants and the legal guardian of the children.

### Participants

Children were recruited from a larger control sample of a study on dyslexia^55^ using behavioural methods and fMRI. Thus, children were already familiar with the MRI apparatus and functioning. Twenty-four children were tested with the LDT paradigm. Seven children were excluded because of strong movement artefacts (see section “Image acquisition and analyses”). This resulted in a sample of 17 children (7 females; 6–9 years; *M* = 7.65; *SD* = 0.86) and to have a balanced data set 17 adults (10 females; 19–30 years; *M* = 24.0 years, *SD* = 3.97). Parents received financial compensation for their travel expenses and children were rewarded with age-appropriate educational gifts. All of the adults’ cohort were psychology students who participated for study credit. No participant had a history of neurological diseases.

### Stimuli

Of each valence category, positive, negative and neutral, 20 words were chosen from the kidBAWL database^32^, where 6–12 years old children rated words regarding valence, arousal and imageability (see in Supplementary, Table A) resulting in a stimulus set of 60 words. These words were matched for arousal, number of letters and syllables over all three valence categories. Word frequencies were taken from the *childLex* database^56^ to ensure similar distributions over all three valence categories (see Supplementary, Table A). In addition, 60 pseudowords (also in Supplementary, Table B) were created based on those chosen words. Words were presented auditorily to avoid potential effects of reading ability. Stimuli were spoken by a female computer voice (MAC OSX voice “Anna”). Each spoken word stimulus lasted 1 s.

The pseudowords were carefully constructed following phonological rules of German^57^. The following three rules were applied to derive them from the 60 words: (1) only the first syllable was changed; (2) consonants were linearly exchanged within articulation mode by changing either articulation location or voicing, as long as this did not violate any phonological rules of the German phonological system; and (3) vowels were altered by changing roundness (e.g., [e:] to [o:], [u:] to [a:], etc.). Coltheart, Besner, Jonasson, & Davelaar^58^ showed interactions in identifying pseudowords and words when a pseudoword is phonologically similar to an existing word (e.g., “brane”). Thus, in the present study such cases were avoided.

### Experimental paradigm

Participants performed the lexical decision task (LDT) during fMRI scanning. In each of the two runs in which the LDT was performed for 5.5 min, 60 stimuli were presented (10 stimuli of each valence category and 30 pseudowords) in an event-related design paradigm. Participants decided as fast and accurately as possible, whether the presented stimulus was a word or pseudoword and indicated their answer via index finger button press. After the 1 s stimulus presentation, participants had 2 s for their response, during which pictures of a pile of books and a magic wand were displayed as response options. The magic wand was chosen to picture the pseudowords because pseudowords were explained as magic words to the participants to make the abstract task easier for the children. Response hand (left vs. right) was balanced over runs to control for motor confounds in the fMRI data. Words were presented in pseudorandomised order where the presentation algorithm controlled that not more than two words or pseudowords were presented consecutively. Inter trial intervals were optimised using Optseq2 algorithm^60^ to *M* = 1000 ms, *SD* = 599 ms, *r* = 500–4500 ms. During the scanner session, participants also performed a valence decision task in additional runs.

### Image acquisition and analyses

The functional data was recorded with a 3 T SIEMENS Tim Trio scanner (SIEMENS Erlangen, Germany) at the Centre for Cognitive Neuroscience Berlin (CCNB). High resolution T1 weighted anatomic reference images were collected as a set of 176 sagittal slices (slice thickness = 1 × 1 × 1 mm, TR = 1.9 s, TE = 2.52 ms, FOV = 256 mm). In both runs 66 functional images were acquired each with a multi echo planar sequence (voxel size = 3 × 3 × 3 mm^3^, TR = 2330 ms, TE1 = 15 ms, TE2 = 34 ms, TE3 = 53 ms, FOV = 192 mm, FA = 70°). In total, the scan procedure took about 24 min. Exact scanner time depended on the individual need for breaks between the runs. The auditory stimuli were presented via circumaural earphones (VisuaStim, MR Research, USA). The response pictures were presented in the middle of the screen with a white background on dual display goggles (VisuaStim, MR Research, USA) using Python 2.7 (Python Software Foundation).

fMRI data analysis was performed using SPM12 (Wellcome Department of Imaging Neuroscience, University College London, UK, 2014). For motion correction, images were realigned to the first image. Next, the ArtRepair toolbox^61^ was used to determine images with scan-to-scan motion parameters over 1.5 mm/TR over global mean^62^. Participants moving more than eleven volumes in a row were excluded. Thus, there were never more than ten consecutive volumes interpolated from preceding and following images. Less than 1.6% of the scan data was repaired over all children. For the children, an age-appropriate segmentation template for 6-year-old children based on Template-O-Matic toolbox^63^ was generated and used for the segmentation of children’s T1 images. Adults’ T1 images were segmented into six tissue probability maps (white, grey, CSF, bone, soft tissue and air). In a next step, a group mean template was generated to enhance comparability within each group within the normalisation preprocessing by the DARTEL algorithm^64^. The functional images were spatially normalised to MNI space and smoothed with an isotropic 8 mm full-width-at-half-maximum (FWHM) Gaussian kernel. Studies supported the feasibility of using adult-defined stereotaxis space for analysis of children older than 6 years^65^. Finally, data was detrended to remove global drifts^66^.

For statistical assessment of activation differences, we used the standard general linear model approach as implemented in SPM. We modelled the trials as regressors in four conditions (pseudowords and three valence categories: positive, negative, neutral). Realignment parameters were included as regressors of no interest. On the first level, we computed baseline contrasts for the four different conditions: the baseline contrast for lexicality ([words (positive, negative and neutral)] > pseudowords), and the affect specific contrasts for the real words (positive > neutral words, negative > neutral words). Group level differences were assessed in second-level ANOVA designs using the flexible factorial design specification of SPM. We first tested for group level effects of the contrast words > pseudowords for adults and children separately, and both cohorts together using a conjunction analysis. Next, we computed a further second-level model to test group level effects for the contrasts positive > neutral words and negative > neutral words for adults and children, respectively. As documented in the Supplementary (Tables C–E), we additionally tested for commonalities of processing positive, negative and neutral words in terms of a conjunction analysis against the conjunction null hypothesis^67^ for both age cohorts separately as well as both age cohorts together. All results are presented p < 0.05 familywise error corrected (FWE) on the cluster level.

On the behavioural level, reaction times were analysed by calculating the mean times between stimulus presentation and button press by one factorial ANOVA and pairwise t-tests between valence categories.

## Supplementary Information

Supplementary Information
