# Supplementary material for: Neural correlates of affective contributions to lexical decisions in children and adults
Source: Sci Rep. 2021 Jan 13;11:945. doi: 10.1038/s41598-020-80359-1 (PMC7806850; doi:10.1038/s41598-020-80359-1)
Supplement: Supplementary file 1 — Supplementary Information [file 41598_2020_80359_MOESM1_ESM.docx]

Neural correlates of affective contributions to lexical decisions in children and adults

Teresa Sylvester*^1,2^, Johanna Liebig^1,2^ and Arthur M. Jacobs^1, 2^

^1^Department of Education and Psychology, Experimental and Neurocognitive Psychology, Freie Universität Berlin, Berlin, Germany

^2^Center for Cognitive Neuroscience Berlin, Freie Universität Berlin, D-14195 Berlin, Germany

**Email addresses of authors:**

Teresa Sylvester: [teresa.sylvester@fu-berlin.de](mailto:teresa.sylvester@fu-berlin.de)

Johanna Liebig: johanna.liebig@fu-berlin.de

Arthur M. Jacobs: [ajacobs@zedat.fu-berlin.de](mailto:ajacobs@zedat.fu-berlin.de)

**Corresponding author**: Teresa Sylvester

Email: Teresa.sylvester@fu-berlin.de

Telephone number: +49 30 838 61829

Postal address: Freie Universität Berlin

Allgemeine und Neurokognitive Psychologie

Habelschwerdter Allee 45

D-14195 Berlin, Germany

| **Valence category** | **Positive** | | **Negative** | | **Neutral** | |  |
| --- | --- | --- | --- | --- | --- | --- | --- |
| **Word property** | ***M*** | ***SD*** | ***M*** | ***SD*** | ***M*** | ***SD*** | ***p*** |
| Valence | 0.87 | 0.39 | -1.35 | 0.47 | 0.28 | 0.31 | <0.001* |
| Arousal | 0.22 | 1.03 | 0.39 | 0.71 | -0.14 | 0.75 | 0.137 |
| Letters | 6.1 | 1.44 | 6.0 | 1.17 | 6.0 | 1.17 | 0.959 |
| Syllables | 1.9 | 0.44 | 1.8 | 0.52 | 1.95 | 0.51 | 0.623 |
| Frequency | 142.04 | 428.99 | 36.39 | 115.27 | 33.47 | 51.31 | 0.326 |

**Table A.** Semantic and lexical variables of presented word stimuli. Valence and arousal rating values are z-transformed (scale -2.5 to 2.5) ranged for positive (r = 0.5 to 2.5), negative (-0.5 to -2.5) and neutral words (-0.5 to 0.5). Frequencies are reported in frequency per million.

| **Positive** | **Negative** | **Neutral** |
| --- | --- | --- |
| Engel (angel) [oerl] | Abfall (rubbish) [otfall] | Ausreden (finish speaking) [euschreden] |
| Ferien (holidays) [sierien] | Angst (fear) [olst] | Arbeit (work) [olbeit] |
| Freizeit (leisure) [schlautzeit] | Begraben (buried) [duegraben] | Arzt (doctor) [orst] |
| Freuen (pleased) [schlauen] | Bestie (beast) [doestie] | Batterie (battery) [dotarie] |
| Freund (friend) [schlaunt] | Betrug (fraud) [duetruk] | Fleisch (meat) [seisch] |
| Frühjahr (spring) [schliehjahr] | Fehlen (lack) [schlielen] | Gummi (gum) [bammi] |
| Fühlen (feeling) [pfuhlen] | Feind (enemy) [seut] | Insekt (insect) [oemsekt] |
| Geschenk (gift) [boschenk] | Fluch (curse) [soch] | Leiter (ladder) [rauter] |
| Gewinn (gain) [bowinn] | Friedhof (cemetery) [schlethof] | Meter (meter) [nater] |
| Harmonie (harmony) [schurmonie] | Gewalt (violence) [bowalt\| | Note (music note) [mite] |
| Herz (heart) [schoerz] | Gift (poison) [boeft] | Prozent (percent) [klozent] |
| Kirsche (cherry) [toersche] | Hassen (hate) [schussen] | Pumpe (pump) [kampe] |
| Küssen (kissing) [tossen] | Killer (ink eraser) [toeller] | Revier (territory) [lavier] |
| Lächeln (smiling) [recheln] | Rache (revenge) [leche] | Ruder (paddle) [leuder] |
| Lob (praise) [raeb] | Sarg (coffin) [ferg] | Sprung (jump) [skung] |
| Mama (mom) [nema] | Scheusal (monster) [feisal] | Suchen (look for) [fachen] |
| Natur (nature) [metur] | Sklave (slave) [spave] | Urteil (decision) [olteil] |
| Party (party) [kerty] | Stehlen (stealing) [weulen] | Winter (winter) [zunter] |
| Triumph (triumph) [plaumph] | Unfall (accident) [omfall] | Zählen (counting) [schoelen] |
| Zukunft (future) [wekunft] | Unheil (mischief) [umheil] | Zwieback (rusk) [jaback] |

**Table B.** Words and pseudo words per valence category Spoken word (its translation) and [spoken pseudoword].

| **Anatomical location** | **MNI** |  |  | **Size** | **Peak** |
| --- | --- | --- | --- | --- | --- |
|  | **x** | **y** | **z** | **k** | **T** |
| **Frontal** |  |  |  |  |  |
| L Pars opercularis | -54 | 16 | -4 | 6054 | 11.92 |
| *L Superior temporal* | -62 | -26 | 0 |  | 10.39 |
| *L Insula anterior* | -38 | 18 | -2 |  | 10.09 |
| L Precentral | -46 | -2 | 52 | 185 | 6.43 |
| *L Middle frontal* | -38 | 4 | 48 |  | 4.73 |
| **Subcortical structures** |  |  |  |  |  |
| R Insula anterior | 36 | 20 | -2 | 4011 | 9.87 |
| R Temporal pole | 56 | 14 | -10 |  | 9.48 |
| R Superior temporal | 62 | -18 | -2 |  | 9.4 |
| **Occipital** |  |  |  |  |  |
| R Calcarine | 14 | -74 | 8 | 1551 | 8.29 |
| *L Calcarine* | -8 | -78 | 8 |  | 6.22 |
| **Supplementary motor area** |  |  |  |  |  |
| L SMA | -2 | 6 | 58 | 1809 | 9.41 |
| *L Dorsal cingulate* | 0 | 24 | 34 |  | 7.9 |
| *R SMA* | 2 | 16 | 46 |  | 7.55 |

**Table C.** Adults’ conjunction analysis for positive, negative and neutral words (FWE corrected, p < 0.05, cluster level). Note. x, y, z = peak coordinates according to MNI stereotactic space, cluster size in voxels, t-values for peaks.

| **Anatomical location** | **MNI** |  |  | **Size** | **Peak** |
| --- | --- | --- | --- | --- | --- |
|  | **x** | **y** | **z** | **k** | **T** |
| **Subcortical structures** |  |  |  |  |  |
| L Insula anterior | -36 | 16 | 0 | 2669 | 9.89 |
| *L Pars orbitalis* | -30 | 28 | -2 |  | 8.95 |
| *L Frontal operculum* | -50 | 18 | -6 |  | 7.88 |
| R Insula anterior | 30 | 26 | -2 | 2207 | 8.72 |
| *R Heschl* | 44 | -24 | 8 |  | 6.43 |
| R Thalamus | 20 | -10 | 14 | 230 | 5.06 |
| *R Caudate* | 20 | 4 | 12 |  | 4.25 |
| **Temporal** |  |  |  |  |  |
| L Superior temporal | -52 | -32 | 6 | 1884 | 8.33 |
| **Parietal** |  |  |  |  |  |
| R Angular | 50 | -60 | 48 | 164 | 5.01 |
| **Occipital** |  |  |  |  |  |
| L Calcarine | -8 | -82 | 14 | 1287 | 8.65 |
| *R Calcarine* | 10 | -80 | 12 |  | 7.56 |
| *R Cuneus* | 10 | -86 | 18 |  | 5.02 |
| **Supplementary motor area** |  |  |  |  |  |
| L SMA | 0 | 4 | 64 | 2083 | 7.73 |
| *R SMA* | 4 | 22 | 38 |  | 7.48 |
| *L Dorsal cingulate* | 0 | 14 | 36 |  | 6.84 |

**Table D.** Children’s conjunction analysis for positive, negative and neutral words (FWE corrected, p < 0.05, cluster level). Note. x, y, z = peak coordinates according to MNI stereotactic space, cluster size in voxels, t-values for peaks.

| **Anatomical location** | **MNI** |  |  | **Size** | **Peak** |
| --- | --- | --- | --- | --- | --- |
|  | **x** | **y** | **z** | **k** | **T** |
| **Frontal** |  |  |  |  |  |
| L Middle frontal | -34 | 48 | 18 | 186 | 5.53 |
| *L Superior frontal* | -24 | 52 | 16 |  | 3.98 |
| **Subcortical structures** |  |  |  |  |  |
| L Insula anterior | -36 | 16 | 0 | 1514 | 9.69 |
| *L Frontal operculum* | -50 | 18 | -6 |  | 7.88 |
| R Insula anterior | 34 | 24 | -2 | 1427 | 8.06 |
| *R Superior temporal* | 60 | -26 | 6 |  | 6.0 |
| **Temporal** |  |  |  |  |  |
| L Superior temporal gyrus | -62 | -24 | 6 | 596 | 7.81 |
| *L Heschl* | -54 | -20 | 6 |  | 5.39 |
| **Occipital** |  |  |  |  |  |
| R Calcarine | 10 | -78 | 10 | 700 | 7.09 |
| L Calcarine | -8 | -78 | 8 |  | 6.22 |
| **Supplementary motor area** |  |  |  |  |  |
| L SMA | -2 | 6 | 62 | 1097 | 7.16 |
| *R SMA* | 2 | 20 | 40 |  | 7.09 |
| *R ACC* | 6 | 32 | 22 |  | 5.48 |

**Table E.** Conjunction analysis for adults and children for positive, negative and neutral words (FWE corrected, p < 0.05, cluster level). Note. x, y, z = peak coordinates according to MNI stereotactic space, cluster size in voxels, t-values for peaks.
